# Bacterial and viral coinfection in idiopathic pulmonary fibrosis patients: the prevalence and possible role in disease progression

**DOI:** 10.1186/s12890-022-01853-y

**Published:** 2022-02-11

**Authors:** Mohsen Moghoofei, Shayan Mostafaei, Nasim Kondori, Michelle E. Armstrong, Farhad Babaei

**Affiliations:** 1 Infectious Diseases Research Center, Kermanshah University of Medical Sciences, Kermanshah, Iran; 2 Department of Microbiology, Faculty of Medicine, Kermanshah University of Medical Sciences, Kermanshah, Iran; 3 Division of Clinical Geriatrics, Department of Neurobiology, Care Sciences and Society, Karolinska Institute, Stockholm, Sweden; 4 Department of Pediatrics, Faculty of Medicine, Kermanshah University of Medical Sciences, Kermanshah, Iran; 5 Department of Clinical Medicine, School of Medicine, Trinity Biomedical Sciences Institute, Trinity College Dublin, Dublin 2, Ireland

**Keywords:** Idiopathic pulmonary fibrosis, Bacterial infection, Viral infection, Coinfection

## Abstract

**Background:**

Idiopathic pulmonary fibrosis (IPF) is a progressive interstitial pneumonia of unknown aetiology with a mean survival rate of less than 3 years. No previous studies have been performed on the role of co-infection (viral and bacterial infection) in the pathogenesis and progression of IPF. In this study, we investigated the role of viral/bacterial infection and coinfection and their possible association with pathogenesis and progression of IPF.

**Methods:**

We investigated the prevalence and impact of bacterial and viral coinfection in IPF patients (n = 67) in the context of pulmonary function (FVC, FEV_1_ and DL_CO_), disease status and mortality risk. Using principal component analysis (PCA), we also investigated the relationship between distribution of bacterial and viral co-infection in the IPF cohort.

**Results:**

Of the 67 samples, 17.9% samples were positive for viral infection, 10.4% samples were positive for bacterial infection and 59.7% samples were positive coinfection. We demonstrated that IPF patients who were co-infected had a significantly increased risk of mortality compared (*p* = 0.031) with IPF patients who were non-infected [Hazard ratio: 8.12; 95% CI 1.3–26.9].

**Conclusion:**

In this study, we report for the first time that IPF patients who were coinfected with bacterial and viral infection have significantly decreased FVC and DL_CO_ (% predicted). Besides, the results demonstrated the increased AE-IPF, increased incidence of death and risk of mortality in infected/coinfected patients compared to non-infected IPF patients.

## Background

Idiopathic pulmonary fibrosis (IPF) is a fatal interstitial lung disease with a mean survival rate of less than 3 years. The prevalence of IPF is estimated at up to 29 cases per 100,000, with the incidence and associated mortality currently increasing [1, 2]. Many risk factors have been implicated in the etiology of IPF including inhaled toxins, smoking and infectious disease [3, 4]. However, the specific mechanisms underlying IPF pathogenesis or disease progression are unknown.

Extensive research has demonstrated a role for a number of viruses in initiation and progression of clinical disease in IPF. Previous research has demonstrated a role of viruses in initiation and progression of clinical disease in IPF [5–9]. Previously, we demonstrated the presence of parainfluenza, RSV, rhinovirus and coronavirus in nasopharyngeal (NPL) and bronchoalveolar lavage (BAL) fluid from IPF patients [4]. However, we did not investigate the impact of these viruses on pulmonary function in IPF patients. The similarities in clinical and radiologic presentation between pneumonitis related to viral infection and acute exacerbation of IPF (AE-IPF) in patients illustrates the key role of viruses in the pathogenesis of IPF [10].

More recently, a role for bacterial infection has been described in the pathogenesis of IPF. Studies have demonstrated that IPF patients have an increased bacterial load in BAL fluid compared with healthy individuals or COPD patients [11, 12]. Furthermore, it has been shown that IPF patients with an increased bacterial load in their BAL fluid at diagnosis have a significantly increased mortality risk [11]. Bacterial species such as *Strepococcus, Veillonella, Haemophillus* and *Neisseria* have been found at increased frequencies the the BAL fluid of IPF patients [11]. Recently, bacterial infection has also been implicated in disease progression during acute exacerbations in IPF (AE-IPF)[13].

In this study, we investigated the prevalence and impact of bacterial and viral co-infection in a cohort of IPF patients (n = 67) in the context of pulmonary function [forced vital capacity (FVC), forced expiratory volume 1 (FEV_1_) and diffusion lung capacity for carbon monoxide (DL_CO_)], disease status and mortality risk. Specifically, we determined the prevalence of: (1) six significant respiratory viral infections in IPF including influenza, parainfluenza, adenovirus, rhinovirus, coronavirus and respiratory syncytial virus (RSV); (2) the prevalence of five respiratory bacterial infections including *Pseudomonas aeruginosa (P. aeruginosa), Streptococcus pneumoniae* (*S. pneumoniae*), *Staphlococcus aureus* (*S. aureus*), *Klebseilla pneumoniae* (*K. pneumoniae*) and *Haemophilus influenza* (*H. influenza*) and (3) we also investigated the incidence of co-infection with bacteria and viruses. Using principal component analysis (PCA), we investigated the relationship between and distribution of bacterial and viral co-infection in the IPF cohort.

## Methods

### Study population

In this retrospective cohort study, 67 nasopharyngeal lavage (NPL) and bronchoalveolar lavage (BAL) samples were collected from IPF patients referred to hospitals of the Kermanshah University of Medical Sciences (KUMS) between June 2017 and September 2018. All acute exacerbated patients were hospitalized. Inclusion criteria were: radiological, spirometry, therapeutic, and biological data, which are considered for IPF patients by the clinical team of KUMS hospitals [2]. Exclusion criteria were: Patients with chronic hypersensitivity pneumonitis, connective tissue disease or asbestosis. The Ethical Committee of KUMS approved this study.

### Nucleic acid extraction

DNA and RNA extraction were performed using 200 ml of NPL and/or BAL specimens by QIAamp MinElute Virus Spin Kit (Qiagen, Hilden, Germany), according to the manufacturer’s instructions. Extracted genomic DNA/RNA was stored at − 80 °C before use.

### DNA array assay for detection of viruses and bacteria

The CLART® Pneumovir DNA array assay (Genomica, Coslada, Madrid, Spain) was used to detect a number of viruses in this study (RSV, influenza viruses, HPIV, rhinovirus, adenovirus and coronavirus) according to manufacturer’s instructions and as described by us previously [4]. Detection of *S. pneumonia*, *S. aureus* and *H. influenza* was also carried out using this method.

### Polymerase chain reaction (PCR) for detection of bacteria

Detection of *P. aeruginosa* was carried out by PCR analysis as described by Tyler et al. previously [14]. Detection of *K. pneumoniae* was confirmed by PCR based on a study by Turton et al. [15].

### Statistical analysis

In this study, all data was presented as the mean ± standard deviation for continuous variables. Categorical variables are presented as N (%). A normality test performed for the continuous variables using Kolmogorov–Smirnov test. A Student’s t-test (parametric) or the Mann Whitney test (non-parametric) was used to test for statistical significance (two-tailed) between two experimental groups. Two-sided Chi square/Fisher’s exact tests were used to assess the associations between IPF and the categorical variables. Principal component analysis was used in order to investigate the pattern of bacterial and virus infections, and coinfection in the patients. Kaplan–Meier survival curve analysis and log rank test were used to test time-to-death between non-infected, and bacterial-, viral- and co-infection groups of IPF patients. False discovery rate (FDR) was corrected using the Benjamini–Hochberg correction method for multiple comparisons. All statistical analysis were analyzed using R software version 3.5.1 and STATA software versions 11.2. Statistical significance was recorded at *p* < 0.05.

## Results

### Study subjects

A final diagnosis of IPF was made after multidisciplinary team discussion; 12 of the 79 recruited patients did not fulfill the American Thoracic Society (ATS) diagnostic criteria for IPF and were subsequently excluded from the study [2]. The remaining 67 subjects with IPF were predominantly men 38 (56.7%) with the mean age 62.8 (SD = 12.44) years. Twelve (17.9%) and 55 (82.1%) of patients were stable and acute exacerbation (AE-IPF). The median time from diagnosis to an acute disease status (AE-IPF) was 90 days. All IPF patients suffered chronic pneumonia, as diagnosed by CT scan in recent years. Nine of 55 (16.3%) of the AE-IPF patients had both fever and myalgia, which was suggested viral-like illness. All patients had moderately severe disease at enrollment as characterized by carbon monoxide diffusing capacity (DL_CO_) (70.5% predicted ± 3.96), Forced Expiratory Volume in 1 s (FEV_1_) (70.9% predicted ± 4.30) and Forced Vital Capacity (FVC) (75.3% predicted ± 4.37). Additional details are included in Table 1.

**Table 1:** Baseline characteristics of the IPF patients (n = 67)

Characteristics	
Age (year)^*^	62.8 ± 12.44
FVC^*^	75.3 ± 4.37
FEV_1_^*^	70.9 ± 4.30
DL_CO_^*^	70.5 ± 3.96
Sex^+^	
Male	40 (59.7)
Female	27 (40.3)
Disease status^+^	
Acute exacerbation	55 (82.1)
(AE-IPF) stable	12 (17.9)
Immunosuppression drugs^+^	
Pred 5 mg	2 (3)
Pred 10 mg	5 (7.5)
Pred 20 mg	2 (3)
CyA 125 mg	2 (3)
Unknown	8 (11.9)
Surgical lung biopsy^+^	
No	63 (94)
Yes	4 (6)
History of fibrosis in family^+^	
No	55 (82.1)
Yes	12 (17.9)
Bacterial infection No. 7 (10.4%)	
*P. aeruginosa*^+^	17 (27)
*S. pneumonia*^+^	17 (27)
*S. aureus*^+^	16 (25.4)
*K. pneumoniae*^+^	16 (25.4)
*H. influenzae*^+^	29 (46)
Viral infection No. 12 (17.9%)	
Parainfluenza^+^	12 (19)
Influenza^+^	12 (19)
Adeno^+^	10 (15.9)
Rhinovirus^+^	25 (39.7)
RSV^+^	15 (23.8)
Coronavirus^+^	9 (14.3)
Co-infection^+^ No. 40 (59.7%)	
Death^+^	
No	49 (73.1)
Yes	18 (26.9)

*Ref.* considered as the reference level for each categorical variable, *NA* not available, forced vital capacity, *FVC* forced expiratory volume 1, *FEV*_*1*_*,*diffusing capacity of lung for carbon monoxide: DL_C0_

^*^Indicated as mean ± standard deviation

^+^Indicated as “n” (%)

### Prevalence of bacterial-, viral- and co-infection rates in IPF patients

Of the 67 samples collected, 12 (17.9%) samples were positive for viral infection, 7 (10.4%) samples were positive for bacterial infection and 40 (59.7%) samples were positive both viral and bacterial infection (coinfection). Among the mono infections, rhinovirus (39.7%) and H. influenza (46%) were detected at the highest rate in IPF patient samples. In contrast, coronavirus (14.3%), *S. aureus* (25.4%) and *K. pneumoniae* (25.4%) were detected at the lowest rate in IPF patient samples. Additional details are included in Table 1.

### Effect of bacterial-, viral- and co-infection on pulmonary function in IPF patients

Here, we carried out an analysis of the effect of viral-, bacterial- and co-infection on pulmonary function in IPF patients. In Table 2, we examined FVC, FEV1 and DLCO indices in IPF patients who were non-infected (Group 1), infected with bacteria only (Group 2), infected with virus only (Group 3) and coinfected with virus and bacteria (Group 4). In this study, there was a significant decrease in FVC values (% predicted) in IPF patients who were coinfected with virus and bacteria compared with patients were non-infected (*p* = 0.013; Table 2). There was also a significant difference in DLCO values (% predicted) in IPF patients who were infected with bacteria (*p* = 0.030; Table 2) or coinfected (*p* = 0.001; Table 2), respectively, compared with non-infected IPF patients. There was no significant difference in FEV1 values (% predicted) in non-infected (Group 1) compared with any of the infected groups of IPF patients (Groups 2, 3 and 4).

**Table 2:** Comparison of pulmonary function indices between non-infected, bacterial-, viral- and co-infected IPF patients, respectively

PFT index	Non-infected *Group 1* (n = 8)	Bacterial Infection *Group 2* (n = 7)	Viral Infection *Group 3* (n = 12)	Coinfection *Group 4* (n = 40)	R, Adj. p*	R, Adj. *p*^+^	R, Adj. *p*^$^
FVC	79.7 ± 3.30	76.6 ± 5.19	77.8 ± 2.88	73.7 ± 4.08	0.961, 0.368	0.976, 0.652	0.924, **0.013**
FEV_1_	73.5 ± 5.0	73.1 ± 5.55	73.1 ± 3.39	69.5 ± 3.82	0.994, 0.996	0.994, 0.992	0.945, 0.152
DL_CO_	76.5 ± 1.29	71.7 ± 4.64	71.2 ± 2.73	69.3 ± 3.70	0.937, 0.076	0.93, **0.030**	0.905, **0.001**

Data represents Mean ± SD

IPF uninfected patients are considered as the reference group (Group 1). The Adj. P is based on the marginally adjusted *p* values by the Benjamini–Hochberg-FDR correction at α = 0.05. Bold values indicated as statistically significant at *p* < 0.05 level

*PFT* pulmonary function test, *R* ratio

^*^Comparison between group 2 versus group 1

^+^Comparison between group 3 versus group 1

^$^Comparison between group 4 versus group 1

### Effect of bacterial-, viral- and co-infection in on disease status, survival status and survival time on IPF patients at 60-month follow-up

**Fig. 1:**
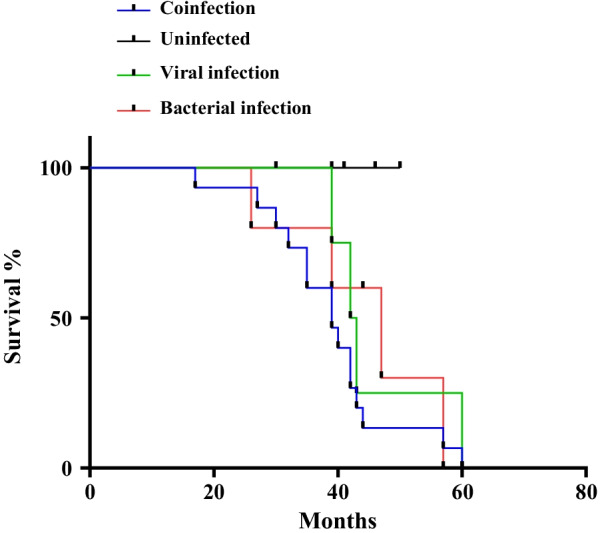
Kaplan–Meier survival curve analysis for comparison of time until death (in months) between uninfected (black line) and bacterial—(red line), viral—(green line) and co-infected (blue line) IPF patients. Coinfected IPF patients had a significantly increased risk of death compared with uninfected patients [Log rank test: *p* = 0.031; Hazard ratio: 8.12; 95% CI 1.3–26.9]

Three important factors including disease status (AE-IPF versus stable-IPF), survival status (death vs. survive) and survival time (months-to-death) were investigated in non-infected, viral infected, bacterial infected and coinfected IPF patients (Table 3). In this study, we demonstrated that IPF patients who were coinfected had more unstable disease, had a higher incidence of death and a short survival time compared with non-infected IPF patients. Specifically, a significantly greater percentage of coinfected IPF patients (55%) were found to be in the AE phase of disease compared with non-infected patients (0%) (*p* < 0.001; Table 3). This suggests that virus and bacterial coinfection led to an increase in the severity of the disease. Investigation of the number of deaths within the IPF study cohort revealed that a significantly greater percentage of coinfected IPF patients (37.5%) died compared with non-infected patients (0%) (*p* = 0.043; Table 3). An investigation of the survival time from diagnosis (months-to-death) in IPF patients demonstrated that survival time in coinfected patients (32.9 ± 9.12 months) was significantly less than survival in non-infected IPF patients (42.5 ± 6.55 months) (*p* = 0.026; Table 3). Furthermore, Fig. 1, using Kaplan–Meier survival curve analysis and the log-rank test, we demonstrated that IPF patients who were co-infected (blue line) had a significantly increased risk of mortality compared (Log rank text: *p* = 0.031) with IPF patients who were non-infected (black line) [Hazard ratio: 8.12; 95% confidence interval (CI) 1.3–26.9].

**Table 3:** Comparison of disease status, survival time and death occurrence between non-infected, bacterial-, viral- and co-infected IPF patients, respectively

Characteristics	Non-infected *Group 1* (n = 8)	Bacterial Infection *Group 2* (n = 7)	Viral Infection *Group 3* (n = 12)	Coinfection *Group 4* (n = 40)	p*	*p*^+^	*p*^$^
Disease status^−^ (acute vs. stable)	0	1 (14.3)	1(8.3)	22 (55)	0.675	0.930	** < 0.001**
Death status^−^ (death vs. survive)	0	2 (28.5)	0	15 (37.5)	0.40	0.622	**0.043**
Survival time^+^ (months-to-death)	42.5 ± 6.55	38.5 ± 7.02	34.4 ± 2.83	32.9 ± 9.12	0.426	0.193	**0.026**

Disease- and death-status are indicated as “n” (%). Survival time is indicated as median ± IQR

IPF uninfected patients are considered as the reference group (Group 1). Bold values indicated as statistically significant at *p* < 0.05 level. Follow up period is 60 months

^*^Comparison between group 2 versus group 1

^+^Comparison between group 3 versus group 1

^$^Comparison between group 4 versus group 1

### Principal component analysis (PCA) of uninfected, viral-, bacterial- and co-infected IPF patients

**Fig. 2:**
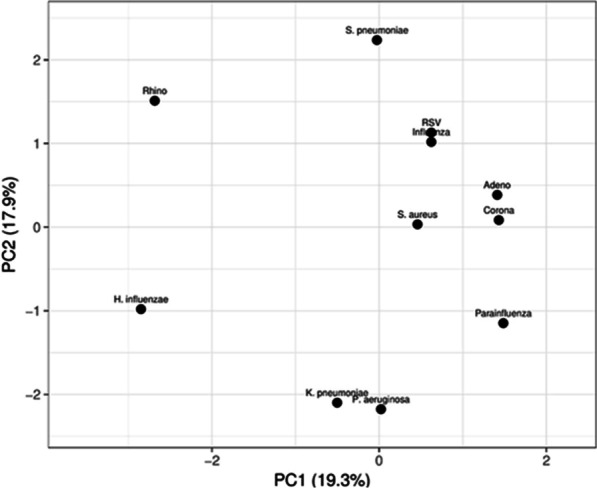
Principal component analysis (PCA) score plot depicting the relationship between bacterial-, viral- and co-infection groups according to PCA based on the first and second principal components for n = 67 IPF patients

Based on Kaplan–Meier curves and log-rank test, we established that IPF patients who are coinfected with virus and bacteria have significantly reduced FVC and DLCO (% predicted), an increased rate of AE-IPF, an increased incidence of death and risk of mortality, and a reduced survival time from months-to-death from diagnosis, respectively, compared with non-infected IPF patients. Here, we employed principal component analysis (PCA), based on the first and second components of the IPF patients included in this study in order to investigate the pattern of bacterial and virus infections, and coinfection in these patients. (Fig. 2). In the PCA score plot, the viral infections (1) RSV and influenza and (2) adenovirus and coronavirus have the most similar coinfection patterns. In the context of bacterial infection, *K. pneumonia* and *P. aeruginosa* have the most similar co-infection pattern.

### Analysis of longitudinal decline in FVC in uninfected, viral-, bacterial- and co-infected IPF patients over a 60-month follow-up period

**Fig. 3:**
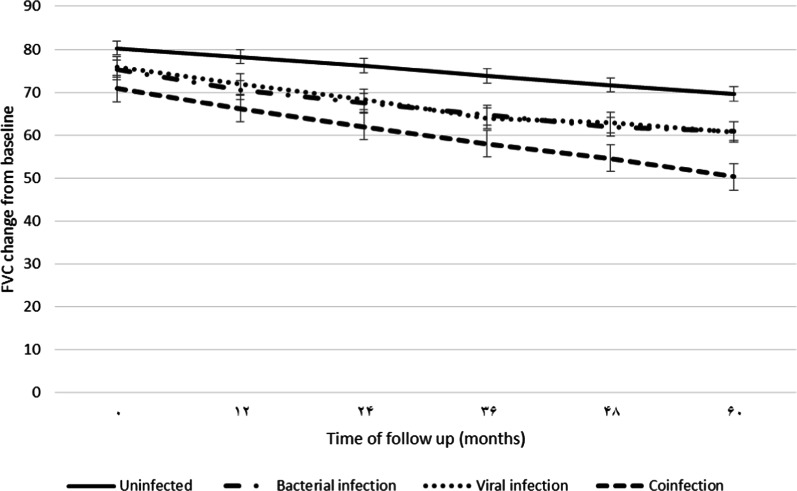
Time-trend analysis of FVC changes from baseline to 60 months after IPF diagnosis. *p* value (uninfected vs. coinfection) < 0.0001. *p* value (uninfected vs. viral infection) < 0.001. *p* value (uninfected vs. bacterial infection) < 0.001. *p* value (viral infection vs. bacterial infection) = 0.679. *p* value (coinfection vs. viral infection) < 0.001. *p* value (coinfection vs. bacterial infection) < 0.001

Analysis of FVC change (% predicted) from baseline was carried out over a 60-month period post-diagnosis as a predictor of IPF disease progression and mortality risk (Fig. 3). We observed a significant decline in FVC change in IPF patients with bacterial infection (*p* < 0.001), viral infection (*p* < 0.001) and co-infection (*p* < 0.0001) compared with uninfected patients. Furthermore, the decline in FVC was significantly greater in co-infected IPF patients compared with patients infected with bacteria (*p* < 0.001) or virus only (*p* < 0.001).

## Discussion

Recent studies have led to the implication of viral or bacterial infections in both the initiation and progression of IPF [5, 11, 13, 16–19]. Previously, viral infection was hypothesized to play a predominant role in the initiation and the progression of IPF [20]. However, more recently a role for bacterial infection has also been implicated in the development of rapidly progressive IPF [11, 13, 18]. Optimal antiviral and antibacterial immunity are vital in the maintenance of lung homeostasis and health in IPF patients.

In this study, we investigated for the first time, the effect of bacterial, viral and co-infection in disease progression in IPF. Here, we demonstrate that IPF patients who are co-infected with bacterial and viral infection has significantly worsened FVC and DL_CO_ function, a greater AE-IPF and reduced survival compared with uninfected patients. Longitudinal rate of decline in FVC (% predicted) is a well-established marker of disease progression and known predictor of mortality in IPF [21, 22]. In this study, these effects were associated with a significantly greater risk of mortality (Hazards Ratio: 8.12; 95% CI 1.3–26.9; *p* = 0.031) using Kaplan Meier survival curve analysis over a period of 60 months follow-up. These results suggest that the status of co-infection in IPF patients may be a good prognostic factor for accelerated disease progression. Additionally, these results suggest that the use of antiviral and/or antibacterial therapies may be useful in treating disease progression in co-infected IPF patients. Currently, clinical trials are underway to investigate the efficacy of the macrolide-type antibiotic, Azithromycin (AZT; ClinicalTrials.gov Identifier: NCT02173145). The antiviral, Valganciclovir, is currently being investigated as an adjuvant therapy with Pirfenidone, in the AE-IPF in patients with a history of CMV infection (ClinicalTrials.gov Identifier: NCT02871401).

AE-IPF are episodes of acute respiratory worsening of unknown cause which may become fatal [13]. Evidence has also suggested that viral infection is responsible for a percentage of acute exacerbations in IPF, which can lead to a rapid deterioration in health [19]. Many patients describe a viral type prodrome before the initial development of respiratory stress in IPF [23]. Currently, there is a growing body of evidence to suggest that bacterial infection, in addition to viral infection, plays a role in AE-IPF [13]. In 2017, Molyneaux et al. demonstrated that there is increased bacterial burden in BAL fluids of IPF patients experiencing AE-IPF compared with stable patients [13]. In this study, we demonstrated that 82.1% IPF patients who were co-infected with bacteria and virus experienced AE-IPF compared with 10.4% patients with bacterial infection only and 17.9% of virus infected patients only. These results suggest a cumulative effect of bacterial and viral infection in the AE-IPF. In order to confirm this, a larger study cohort would be needed.

Using PCA analysis in this study, we demonstrated the following co-infection patterns in IPF patients NPL and BAL fluid samples: (1) coronavirus, parainfluenza virus and adenovirus, (2) rhinovirus and *S. pneumonia*, (3) *H. influenza*, *K. pneumonia* and *P. aeruginosa*, (4) RSV, Influenza and *S. aureus* have similar co-infection patterns. The co-infection pattern of virus and bacteria is of particular interest. Previous studies have shown that viral infection can predispose to bacterial superinfection. Bacterial superinfection of the lung during influenza infection promotes severe disease pathogenesis and leads to increased mortality [24]. Influenza infection can also predispose individuals to *S. aureus* superinfection [25], which is a co-infection pattern observed in our IPF samples. The ability of influenza to facilitate bacterial superinfection in IPF patients underlines a mechanism by which bacterial and viral co-infected IPF patients may experience increased disease progression, mortality risk and reduced lung function as observed in this study.

We previously established that toll-like receptor 3 in an important protective factor against rapid disease progression in IPF [26, 27]. TLR3 is a member of the toll-like receptor superfamily of pathogen recognition receptors (PRRs) [28]. It has previously been shown to bind dsRNA from viruses, bacteria and helminths, respectively, in addition to mRNA released from necrotic cells [29–32]. Recently, our laboratory demonstrated that the TLR3 SNP, Leu412Phe (*TLR3* L412F, rs3775291), which results in defective TLR3 function, is associated with a significantly greater risk of mortality and an accelerated rate of decline in FVC of lung function in IPF patients [26, 33]. Our recent data demonstrates that 412F-heterozygous IPF patients have reduced responses to viral dsRNA and a number of bacterial agonists [26]. We suggest that bacterial and viral co-infection will have a much more deleterious effect in IPF patients who have defective TLR3 function, and are 412F-heterozygous.

In this study, we used NPL and BAL fluid in our to quantitate bacterial and viral infection in IPF patients. However, IPF is a lung disease which affects the parenchymal tissue. In order to assess the level of co-infection in lung tissue, it would be necessary to perform a video-assisted thoracic surgery (VATS) biopsy on patients. These biopsies are associated with considerable risk for the patient and have appreciable rates of morbidity and mortality. Therefore, analysis of co-infection in parenchymal IPF lung is not a viable option. However, it is promising to note that quantitation of levels co-infection in distal IPF samples, such as NPL and BAL fluid, can give significant results which are linked to accelerated disease progression, increased AE-IPF and increased mortality risk. Our study is not without limitations. The main limitations of this study are its retrospective nature and small sample size. The retrospective nature of this study limiting our ability to control for potential confounding factors. Also, low sample size in studied groups could be effect on power of the statistical tests.

## Conclusion

In summary, our results demonstrated that the coinfection is significantly associated with an enhanced risk of death by AE in IPF. Furthermore, this study reveals bacterial and viral co-infection as novel prognostic marker in the treatment of IPF. Further analysis is necessary in order to confirm these findings in a larger cohort of IPF patients.

## Data Availability

The datasets used and/or analyzed during the current study could become available through the corresponding author on reasonable request.

## Abbreviations

IPF: Idiopathic pulmonary fibrosis
PCA: Principal component analysis
AE-IPF: Acute exacerbations in IPF
NPL: Nasopharyngeal lavage
BAL: Bronchoalveolar lavage
FVC: Forced vital capacity
FEV1: Forced expiratory volume 1
DLCO: Diffusion lung capacity for carbon monoxide
RSV: Respiratory syncytial virus
